# Greater temporal regularity of primary care visits was associated with reduced hospitalizations and mortality, even after controlling for continuity of care

**DOI:** 10.1186/s12913-023-09808-7

**Published:** 2023-07-20

**Authors:** Maram Khazen, Wiessam Abu Ahmad, Faige Spolter, Avivit Golan-Cohen, Eugene Merzon, Ariel Israel, Shlomo Vinker, Adam J. Rose

**Affiliations:** 1 Braun School of Public Health and Community Medicine, Faculty of Medicine, Hebrew University of Jerusalem, Ein Kerem Campus, Jerusalem, Israel; 2 Department of Health Systems Management, The Max Stern Yezreel Valley College, Yezreel Valley, Israel; 3 Leumit Health Services, Research Institute, Tel Aviv, Israel; 4 Sackler School of Medicine, Tel Aviv, Israel; 5 Adelson School of Medicine, Ariel University, Ariel, Israel

**Keywords:** Temporal regularity of care, Primary care, Outcomes research, Chronic disease care

## Abstract

**Background:**

Previous studies have shown that more temporally regular primary care visits are associated with improved patient outcomes.

**Objective:**

To examine the association of temporal regularity (TR) of primary care with hospitalizations and mortality in patients with chronic illnesses. Also, to identify threshold values for TR for predicting outcomes.

**Design:**

Retrospective cohort study.

**Participants:**

We used data from the electronic health record of a health maintenance organization in Israel to study primary care visits of 70,095 patients age 40 + with one of three chronic conditions (diabetes mellitus, heart failure, chronic obstructive pulmonary disease).

**Main measures:**

We calculated TR for each patient during a two-year period (2016–2017), and divided patients into quintiles based on TR. Outcomes (hospitalization, death) were observed in 2018–2019. Covariates included the Bice-Boxerman continuity of care score, demographics, and comorbidities. We used multivariable logistic regression to examine TR’s association with hospitalization and death, controlling for covariates.

**Key results:**

Compared to patients receiving the most regular care, patients receiving less regular care had increased odds of hospitalization and mortality, with a dose–response curve observed across quintiles (*p* for linear trend < 0.001). For example, patients with the least regular care had an adjusted odds ratio of 1.40 for all-cause mortality, compared to patients with the most regular care. Analyses stratified by age, sex, ethnic group, area-level SES, and certain comorbid conditions did not show strong differential associations of TR across groups.

**Conclusions:**

We found an association between more temporally regular care in antecedent years and reduced hospitalization and mortality of patients with chronic illness in subsequent years, after controlling for covariates. There was no clear threshold value for temporal regularity; rather, more regular primary care appeared to be better across the entire range of the variable.

**Supplementary Information:**

The online version contains supplementary material available at 10.1186/s12913-023-09808-7.

## Background

Encouraging effective preventive care is a key challenge for health systems [1, 2]. While health organizations have long recognized the importance of delivering proactive and planned care which anticipates and prevents problems rather than merely reacting to them once they occur, until recently there have been few efforts to measure this construct. Recently, researchers have started to examine the temporal regularity of primary care visits as a measure of their likelihood of allowing proactive rather than reactive care [3, 4].

Temporal Regularity (TR) of primary care visits adds an additional dimension to the longstanding concept of continuity of care (COC). COC focuses on the extent to which a patient sees the same primary care physician repeatedly, as opposed to seeing other physicians [5, 6]. There are several measures of COC, all of which have shown to predict better outcomes for patients with higher COC [7–10].

**Fig. 1:**
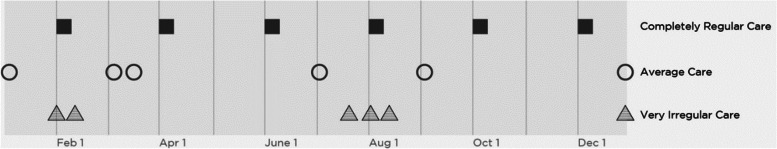
Illustration of the concept of temporal regularity of primary care visits. In the example, there are three patients, each with six visits over the course of one year. They exhibit completely regular care, average care, and very irregular care

More recently the concept of Temporal Regularity (TR) of primary care visits has arisen [11, 12]. TR focuses on a temporal pattern of primary care visits that is regularly spaced in time. For example, two patients might each see a primary care physician six times in a year. However, one might see the physician precisely every 60 days (completely regular care) and the second might have much more irregular spacing between visits (Fig. 1). Regular visits are also more likely to feature proactive care, whereas the irregular visits are more likely to address urgent complaints. Hence, the first patient will probably receive more of the sort of high-value care that will generate better health outcomes.

Over the past decade, several studies have shown a modest but consistent benefit for patient outcomes from more temporally regular primary care appointments, mainly among patients with significant chronic conditions [13, 14]. However, the existing literature on TR has several key limitations. First, it is focused on specific populations, mainly Australia and the United States [4–6, 14]. Second, previous studies have not controlled for COC when analyzing TR, which is important since higher TR and higher COC are likely to be correlated. Third, previous studies have not examined threshold values above or below which TR can prove to be beneficial [15, 16]. Finally, previous studies have not adequately examined which subgroups (e.g., by age, sex, comorbid conditions) are most sensitive to changes in TR [17, 18]. Understanding threshold values and high-risk populations could help us better target TR-related interventions to patients most likely to benefit [19, 20].

In this study, we examined the association of TR with two health outcomes (hospitalizations and mortality) in a population of patients treated by a large health maintenance organization (HMO) in Israel. Unlike previous studies, this one controlled for COC, examined threshold values for TR, and analyzed the disparate impact of TR in key subgroups. The results of this study have the potential to advance our understanding of TR and how it operates in different settings and subpopulations.

## Methods

### Study design

This study is based on data from the electronic health record of Leumit Health Services (LHS), one of four health maintenance organizations (HMOs) in Israel. All four of Israel’s HMOs provide a universal standard basket of services based on the 1995 National Health Insurance Law, according to which every resident is entitled to healthcare services and must belong to one of these HMOs [21, 22]. While a patient is ensured and cared for by an MHO, he or she can only receive subsidized healthcare services including hospitalizations through that HMO. Patients can decide to change their affiliation to another HMO, but the rate of switching between HMOs is quite low, less than 1% per year. Our data consist of a four-year period (2015–2019), chosen in part to avoid the COVID period, when care would not have been delivered in usual ways. In total, the LHS database contained 331 clinics. All patients were assigned to a primary clinic in the LHS database, based on their selection of a PCP. The study was approved by the LHS research ethics committee.

### Participants’ inclusion and exclusion criteria

Participants were members of LHS during the four-year period, including those who died during the period. They were aged 40 years and above and had at least one of the following three chronic conditions: diabetes mellitus, congestive heart failure (CHF), or chronic obstructive pulmonary disease (COPD). These are particularly “significant” chronic conditions, which would have a real impact on the patient’s health [23, 24] and are prevalent among poor and minority populations in Israel [25, 26]. We focused on these conditions since they are prominent in Israel and are a leading cause for hospitalizations [27, 28]. These conditions are among the leading causes of hospitalization in Israel [27, 28]. Also, HMOs have developed structured pathways to address these conditions, especially for diabetes.

Patients who received dialysis were excluded since they visit a doctor at least once a week at dialysis. Therefore, the concept of regularity of care may not apply to them. Also, we excluded participants who died in 2016 or 2017, because we measured all outcomes in 2018–2019, and we could not measure outcomes for those patients. We also limited our dataset to patients who had at least 3 primary care visits during 2016–2017 since it is not possible to characterize TR with fewer than 3 primary care visits.

### Primary care visits

Primary care visits were defined as a clinical encounter with the primary care physician either personally or virtually (e.g., video-link, telephone, or asymmetric communication- text messages), excluding visits to specialists. Primary care visits are clearly identified in the LHS database system because all physicians are labeled as being primary care physicians or specialists. Further, informed by two of the study authors (AJR and YM), who have worked as primary care physicians, we included primary care visits that lasted more than five minutes, a minimal time indicating proactive or comprehensive care and suggesting that the encounter included more than refilling prescriptions. The duration of each encounter was calculated according to the time the physician had the patient’s chart open, which indicates that the patient-physician encounter lasted at least that long.

### Independent variable: temporal regularity of care

TR was calculated based on the number of days between primary care visits. For each patient, we calculated the mean interval between visits (in days), and the standard deviation of that mean. We then divided the standard deviation by mean (i.e., a Coefficient of Variation, or CoV). CoV is a unitless measure that should allow comparisons between patients with different underlying absolute frequencies of care (e.g., between patients who see the doctor twice a year and ten times a year). Because of how CoV is constructed, a higher TR score means less temporally regular care, while a TR score of 0 means that the patient had completely regular care (i.e., exactly the same interval between visits). Throughout this manuscript, for clarity, we refer to more- and less-regular care. In Fig. 1, we show an illustrative example of three patients, each of whom has six visits over the course of a year. One of the patients had very regular care, the second had average care, and the third had very irregular care.

Measuring TR during the same period as the study outcomes has the potential to introduce endogeneity, because being hospitalized or having a morbid illness may result in less regular patterns of care (rather than the reverse). Therefore, consistent with other studies [4, 14], we measured TR in an earlier period and outcomes in a later period, which establishes temporal precedence, meaning that the exposure clearly occurred before the outcome. Establishing temporal precedence is one way to support causal inference [29]. TR was computed during the years 2016–2017, while outcomes were measured during the following two years (2018–2019). Since one of the main aims of this study was to identify threshold values for TR for predicting outcomes, we divided patients into quintiles on TR, and analyzed these quintiles as a class variable.

### Dependent variables: health outcomes

Health outcomes were measured during the years 2018–2019. There were two main outcomes. The first outcome was hospitalizations, defined as any instance of an overnight stay in the hospital as long as it could have been preventable with good primary care. For example, we excluded hospitalizations related to trauma and burns, pregnancy, or rehabilitation. The second outcome was mortality, including date of death.

### Covariates

We applied a list of diagnosis codes to define covariates (Table 1) as has been done by other researchers using LHS data [30–33]. LHS utilizes International Classification of Diseases, Clinical Modification, version 9 (ICD-9) codes for physical health conditions, and ICD-10 codes for mental health conditions. Comorbid conditions were defined according to at least one appearance of any of the codes on this list during 2016–2019.

**Table 1:** Diagnosis codes used to define chronic health conditions

Physical Health Conditions	ICD-9 Codes
Atrial Fibrillation	427.3x
Cancer	140–239, **BUT NOT**: 173, 290.40–209.9x, 210–224, 226–229, 232
Chronic Lung Disease^a^	491.x, 492.x, 494.x, 495.x, 496.x, 500–505
Coronary Artery Disease, Angina	413.x
Coronary Artery Disease, History of Myocardial Infarction	412.x
Dementia or Pre-Dementia	331.x
Diabetes Mellitus^a^	250.x, 357.2, 362.0, 366.41
Epilepsy	345.x
Heart Failure^a^	398.91, 402.01, 402.11, 402.91, 404.01, 404.03, 404.11, 404.13, 404.91, 404.93, 425.x, 428.x
Hypertension	401.x, 402.x, 403.x, 404.x, 405.x
Inflammatory Bowel Diseases	555.x, 556.x
Osteoporosis	733.0x
Peripheral Arterial Disease	440.x, 441–442, 443.89, 443.9
Rheumatoid Arthritis	714.0
Sleep Disorders	327.x **BUT NOT** 327.35
History of Stroke	438.x
Venous Thromboembolism	415.x, 453.x
Mental Health Conditions	ICD-10 Codes
Alcohol Misuse	F10.x **but not** F10.11, F10.21
Anxiety Disorders	F41.x
Attention Deficit Hyperactivity Disorder	F90.x
Bipolar Disorder	F31.x
Depression	F32.x, F33.x
Post Traumatic Stress Disorder	F43.1
Schizophrenia	F20.x, F25.x

*ICD-9* International Classification of Diseases Code, Clinical Modification, Version 9, *ICD-10* International Classification of Diseases Code, Clinical Modification, Version 10

^a^Having at least one of these conditions was an inclusion criterion for being in the study

Instead of using diagnosis codes, chronic kidney disease was assessed directly using estimated glomerular filtration rate (eGFR), computed using the patient’s highest creatinine value in 2016–2017. For missing eGFR, we tried imputing the mean, imputing the mode, and multiple imputation. These choices did not impact the results of our models, so we imputed the mode (60 + , or intact kidney function).

Sociodemographic data were characterized as follows. Ethnic/social groups included Arabs, Ultra-Orthodox Jews, and the “General Population” (all others). Age was based on the year of birth and was divided into the following categories: 40–49, 50–59, 60–69, 70–79, 80–89, and 90 + . Sex was male or female. Patients were identified as living in one of the four regions of Israel: Central (including Tel Aviv), Jerusalem and its surroundings, the South, and the North. Area socioeconomic status (SES) was provided by the POINTS company [34] and is divided into ten levels, from the poorest (1) to the wealthiest (10). The SES index includes measures of the social and economic level in four areas – demographics, education, standard of living, and employment (financial income, motorization level, housing characteristics). We grouped SES into four groups: 1–3 (poorest), 4–5, 6–7, and 8–10 (wealthiest). Patients in group 1–3 were in the lowest socio-economic level, characterized by the lowest level of average income per standard person, and had the least educational attainment.

Further, an important feature in this study was measuring performance of TR while controlling for COC. It is already known that higher COC predicts better health outcomes [7–10]. As stated above, previous studies of TR have not controlled for COC as a covariate [17]. COC was measured according to Bice-Boxerman index [35]. An index of 0 indicates that the patient saw different primary care physicians at all visits (low continuity) and an index of 1 indicates the same caregiver at all visits (high continuity).

### Statistical analyses

We began with univariate analyses of all variables, including TR, the outcomes, and the covariates. We performed bivariate analyses to examine the unadjusted association of TR on the dependent variables, and to test the association between TR and examine relationships between covariates, and between covariates and the outcomes. We then proceeded to multivariable logistic regression, to test linear trends and the association between TR and the study outcomes, controlling for covariates. For a test of linear trend, one sets up the independent variable (here, TR) as an ordinal variable, and then performs a linear regression to test whether the effect of the ordinal variable is statistically significant. Showing significance indicates that the effect of the independent variable on the dependent variable is linear or can be fit as a linear effect.

We also performed stratified subgroup analyses, to see if the association of TR with the outcomes would be the same across groups. Subgroups included: 1) Age groups; 2) Sex; 3) Ethnic/social groups; 4) Socioeconomic levels; 5) patients experiencing certain comorbid conditions (depression, moderate to severe kidney disease, cancer).

In addition to logistic regressions, which simplified hospitalization to a binary variable, we also analyzed it as a count variable, since patients can be hospitalized more than once. For these analyses, we conducted several different regressions to ensure that our methodological choices were not impacting the outcome. We used a Poisson regression, both with all patients included, and after excluding all patients who died during the study (since they had unequal follow up time, violating a key model assumption). We also used a negative binomial model, which is also used for count data, and is less dependent on statistical assumptions [29].

Analyses were conducted using the R statistical package, version 4.1.2.

## Results

### Description of study population

**Fig. 2:**
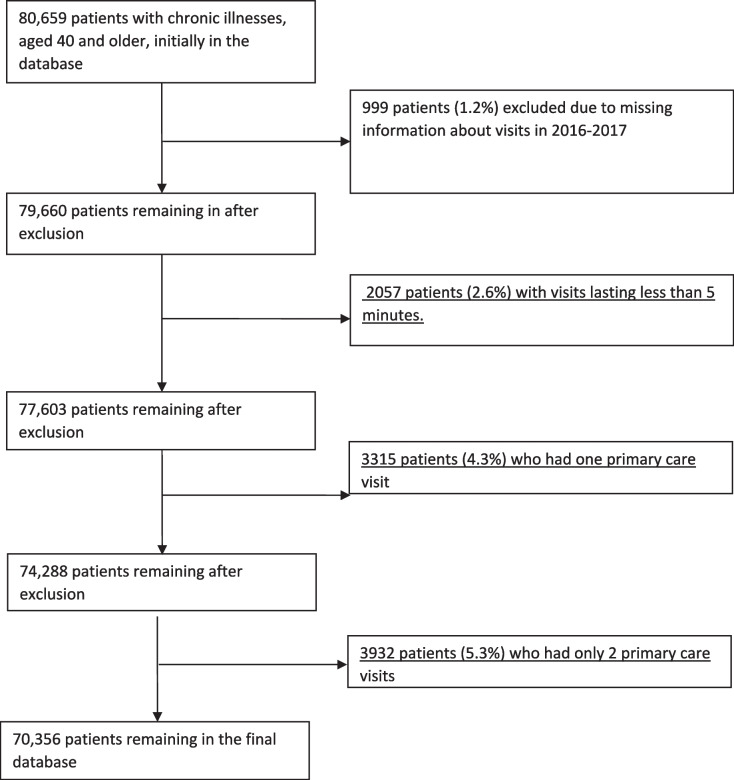
Final database of patients after different stages of exclusion

The study included 80,677 patients, which was reduced to 70,356 after excluding those with less than 3 primary care visits in 2016–2017 (Fig. 2). Patient characteristics are presented in Table 2. Most patients were of lower or lowest SES, and half were female. The majority (59%) were between ages 50–69. Most (77%) were from the general population, while less than a fifth (16%) were Arabs and 6% were ultra-Orthodox Jews. The most common chronic condition was diabetes mellitus (78%).

**Table 2:** Baseline characteristics of the included patients (*n* = 70,356)

Variables	Number	Percent
Age Groups (year born)		
40–49 (1966–1975)	8615	12
50–59 (1956–1965)	18,010	26
60–69 (1946–1955)	22,773	32
70–79 (1936–1945)	13,667	19
80–89 (1926–1935)	6,413	9.1
90 + (1910–1925)	878	1.2
Sex		
Male	35,055	50
Female	35,301	50
Ethnic/cultural group		
Arab	11,288	16
Ultra-Orthodox Jews	4,162	5.9
Others	54,411	77
Region of Israel		
North	18,276	26
Central	21,285	30
Jerusalem	9,195	13
South	21,600	31
Socioeconomic Status (SES)		
Highest	5,921	8.4
Higher	22,124	31
Lower	30,419	43
Lowest	11,270	16
Physical Health Conditions		
Atrial Fibrillation	8,837	13
Cancer	7,908	11
Chronic Lung Disease	20,355	29
Coronary Artery Disease, Angina	20,529	29
Coronary Artery Disease, MI	7,697	11
Dementia/Pre-Dementia	887	1.3
Diabetes	55,089	78
Epilepsy	654	0.93
HF	12,122	17
Hypertension	32,981	47
Inflammatory Bowel Disease	1,239	1.8
Osteoporosis	14,757	21
Peripheral Arterial Disease	7,715	11
Rheumatoid Arthritis	1,986	2.8
Smoking, Current	13,481	19
History of Stroke	3,471	4.9
Venous Thromboembolism	3,800	5.4
eGFR (lowest one recorded)		
60 +	53,830	77
45–59	7,961	11
30–44	3,871	5.5
< 30	1,891	2.7
Mental Health Conditions		
Alcohol Misuse	221	0.31
ADHD	1,281	1.8
Anxiety Disorders	21,087	30
Bipolar Disorder	1,022	1.5
Depression	20,200	29
PTSD	1,790	2.5
Schizophrenia	1,386	2.0

### Distribution of temporal regularity score

TR scores ranged from 0.00 (completely regular care) to a high of 3.57 (extremely irregular care). The median TR was 0.93 and the mean was 0.95 (SD 0.32). We divided patients into quintiles on TR and used these quintiles as the independent variable for all analyses. The mean number of primary care visits of chronically ill patients was 13.5 (SD 10.0) and the median was 11 primary care visits for the baseline period. Similar distributions were observed in the follow-up period (mean 13.6 visits per patient, SD 10.2) and median of 11. We did observe that primary care utilization varied by TR quintile. For example, patients in the first quintile (least temporally regular care) had 9.9 visits (SD 7.9) with a mean of 60.5 (SD 55.9) days between primary care visits, while patients in the second and third quintiles had more visits [14.9 (SD 10.5) and 15.5 (SD 11.0), respectively] with fewer days between the visits [42.6 (SD 46.9) and 40.9 (SD 50.5), respectively]. Patients in the fourth and fifth quintiles had somewhat fewer visits than those in the third quintile, although more than those in the first [14.7 (SD 10.4), 12.3 (SD 8.8), respectively].

### Distribution of continuity of care score and correlation with TR

We calculated the Bice-Boxerman continuity of care score for all patients. COC is between 0 (the patient never saw the same primary care doctor twice) to 1 (the patient only saw one primary care doctor). The mean COC score was 0.65 (SD 0.29), and the median was 0.67 (IQR 0.40, 1). Approximately 25% of patients had a COC score of 1 (only one doctor). COC was divided into four groups of roughly equal size, with all patients with COC of 1 in a single group.

We examined the Pearson correlation between TR and COC to examine how strongly they were related. There was a weak correlation between the two variables (*r* = -0.10, *p* < 0.001). Because the polarity of TR is opposite that of COC, this suggests that patients with greater continuity of care by the same primary care physician also tended to have slightly more temporally regular care. However, the weak correlation also suggests that TR and COC represent separate constructs.

### Temporally irregular care predicting hospitalizations

We examined the association of temporal regularity with hospitalizations (Table 3). Of the 70,356 patients in the sample, 20,51 4(29%) were hospitalized at least once. Compared to patients receiving the most regular care (TR between 0–0.7), a monotonic increase in the probability of hospitalization was observed across quintiles (*p* for linear trend < 0.001). For example, patients with the least regular care had an Odds Ratio -Adjusted (AOR) of 1.24, compared to the reference category. When analyzed as a class variable, all four levels of more-regular care had significantly lower incidence of hospitalization than the reference category. A test of linear trend was statistically significant (*p* = 0.002), meaning that more-regular care was associated with lower levels of hospitalization across all 5 levels of the variable.

**Table 3:** Association between temporal regularity of primary care visits and hospitalizations, using logistic regression. TR was measured over a two-year period (2016–2017) and hospitalizations over the following two years (2018–2019). Total *n* = 70,356 (of whom, 20,514 with at least one hospitalization)

	Range of TR	Percent Hosp. at least once	Odds Ratio – Unadjusted	95% CI Unadjusted	Odds Ratio -Adjusted^a^	95% Adjusted
Quintile 1 of TR (most regular)	0–0.70	24.9	REF		REF	
Quintile 2	0.70–0.85	30.1	1.30 ‡	[1.23,1.37]	1.11 ‡	[1.05,1.18]
Quintile 3	0.85–0.99	30.8	1.34 ‡	[1.27,1.41]	1.16 ‡	[1.09,1.23]
Quintile 4	0.99–1.18	30.7	1.34 ‡	[1.27,1.41]	1.20 ‡	[1.14,1.28]
Quintile 5 (least regular)	1.18–3.19	29.4	1.26 ‡	[1.19,1.33]	1.24 ‡	[1.17,1.31]

^a^Adjusted for all variables in Table 2 (age, sex, sector, region, SES, comorbid conditions), and for Bice-Boxerman continuity of care

^‡^ *p* < 0.001

### Temporally irregular care predicting mortality

We examined the association of temporal regularity with all-cause mortality (Table 4). Of 70,356 patients in the sample, 3,766 died during 2018–2019 (5.4%). Compared with patients with the most regular care, patients with less temporally regular care in 2016–2017 were more likely to die in 2018–2019, after adjusting for covariates. The association was again monotonic across quintiles of TR (*p* for linear trend < 0.001). For example, patients with the least regular care had an AOR of 1.40, compared to the reference category.

**Table 4:** Association between temporal regularity of primary care visits and mortality, using logistic regression. TR was measured over a two-year period (2016–2017) and mortality over the following two years (2018–2019). Total *n* = 70,356 (of whom, 3,766 died)

	Range of TR	Percent mortality	Odds Ratio – Unadjusted	95% CI Unadjusted	Odds Ratio -Adjusted^a^	95% CI Adjusted
Quintile 1 of TR (most regular)	0–0.70	4.39	REF		REF	
Quintile 2	0.70–0.85	5.73	1.32 ‡	[1.19,1.47]	1.17 †	[1.04,1.32]
Quintile 3	0.85–0.99	5.52	1.27 ‡	[1.14,1.42]	1.15 †	[1.02,1.29]
Quintile 4	0.99–1.18	5.51	1.27 ‡	[1.14,1.41]	1.21 †	[1.08,1.36]
Quintile 5 (least regular)	1.18–3.19	5.61	1.29 ‡	[1.16,1.44]	1.40 ‡	[1.24,1.57]

^a^Adjusted for all variables in Table 2 (age, sex, sector, region, SES, comorbid conditions), and for Bice-Boxerman continuity of care

^†^*p* < 0.05

^‡^*p* < 0.001

### Sensitivity analysis: poisson and negative binomial regression for hospitalization

Unlike mortality, hospitalization can occur more than once. Therefore, we used Poisson regression and negative binomial regression to examine the association of TR with hospitalizations, measured as a count variable. The results were generally similar to the main analysis of hospitalizations, and are found in the Online Appendix A.

### Stratified analyses

We repeated the main analyses (logistic regressions of hospitalization and mortality) stratified by age (Table A1-A6), sex (Table B1, B2), ethnic group (Table C1-C4), area-level SES (Table D1-D8), and certain comorbid conditions (Table E1-E6). Results are shown in Online Appendix B. Analyzing smaller groups, in many cases, made the estimates unstable and eroded statistical power to show differences. However, the stratified analyses did not show strong differential association of TR across groups.

## Discussion

In this study, chronically ill patients with more temporally regular care in antecedent years were less likely to be hospitalized or die in subsequent years, after adjusting for covariates. This concords with several previous studies [4, 36, 37] and emphasizes that more temporally regular care appears to be beneficial for patient outcomes, specifically patients suffering from chronic illnesses. Although the significant association of TR with outcomes was modest, it was dose-dependent, which argues for it being real [29, 38]. This study adds Israel to the list of environments where more temporally regular primary care appears to be associated with better health outcomes. It appears, based on our analyses, that more temporally regular care is better across a wide range of values, with no clear threshold value.

Previous studies of TR have not controlled for COC. In our study, TR and COC were weakly correlated, so it is not surprising that controlling for COC did not have a large impact on TR’s association with health outcomes. Our study implies that seeing the same doctor consistently, and seeing a doctor at regularly spaced time intervals, are both beneficial and can promote effective care – however, they are different.

We also looked for important differences between groups of chronically ill patients regarding TR being significantly association with reduction in probability of hospitalization and mortality. We initially had expected that patients from low SES neighborhoods, or those of minority groups, due to being more vulnerable, might benefit more from temporally regular care. We did not find these sorts of differences, suggesting that more temporally regular primary care improved health outcomes, regardless of patients’ SES or other measured outcomes.

This study does not conclusively answer the question of how temporally regular care contributes to better outcomes of patients experiencing chronic illnesses in subsequent years [4, 39]. It seems likely that more temporally regular care is a proxy for more proactive and less reactive care [34, 40]. The ability to provide proactive and high-value care may depend on having regular appointments as opposed to waiting for acute, but ultimately less important, issues to arise as a reason to seek care. However, no study has conclusively demonstrated that this is the mechanism whereby better TR is associated with improved outcomes of chronically ill patients in subsequent years. It also remains to be demonstrated how one would intervene to increase TR, if one wanted to do so. It may be that an effort to intentionally promote TR would give insight into its benefit. It is even possible that higher TR is merely a marker of high-quality care, which in turn leads to better outcomes, but is not the cause of improved outcomes.

Strengths of this study include the complete and detailed longitudinal follow-up data over 4 years, with very little migration of chronically ill patients in or out of the database. However, several limitations should be noted. Subgroup analyses may not have been large enough to produce stable estimates in some cases. Our observational study design limits causal inference, although some aspects of our study (clear temporal precedence, dose–response curve) argue that the findings are real [29]. Another potential limitation relates to TR calculations based on two-year data and having at least three visits to populate the variable, which could leave out some patients who do not have enough visits. This may limit the generalizability of our results to patients with very infrequent primary care visits, although patients with these particular chronic conditions would usually be expected to visit more frequently. Also, because quality measures are followed up annually, calculating TR could prove to be complicated, because requiring two years of data may erode the timeliness and actionability of TR feedback. Also, it is important to acknowledge that our TR calculation did not account for physician-level clustering, although we did account for clinic-level clustering.

Another limitation relates to unmeasured factors that may lead a patient to follow-up when requested to do so. Such a factor, which might be called “taking responsibility for one’s own healthcare”, could also be associated with other behaviors that improve health. We plan to examine this issue in greater detail in our forthcoming qualitative study about TR.

While this study and others have shown a modest, but consistent, benefit to more temporally regular care [4, 14, 41, 42], we clearly have a lot to learn regarding how clinicians and other clinic staff can work to promote higher TR, specifically in patients experiencing chronic illnesses. In a forthcoming study, we will use a qualitative approach to examine how and why some clinics attending to chronic ill patients achieve higher TR than others, after accounting for differences in the patient population. However, our findings here suggest that policy makers could employ strategies promoting better TR of care, whether by special reimbursement to family doctors, nurses, and administrative staff, specific information technology infrastructures enabling follow ups, physicians’ participation in group quality improvement, or by educational programs and pro-health messages targeted to patients [43–49].

In summary, our study found a modest but consistent association between higher TR and improved patient outcomes in subsequent years, adding to a body of literature showing similar findings [4, 40, 41]. While the study was observational, features of the study such as clear temporal precedence and a dose–response curve argue that more temporally regular primary care is beneficial and significantly modifies health outcomes of patients with consequential chronic conditions. This association between TR and patient outcomes (hospitalization, mortality) persisted after controlling for COC. Going forward, it will be important to explore mechanisms by which TR would improve health outcomes and how healthcare systems could intentionally promote temporally regular care.

## Supplementary Information

**Additional file 1. **

## Data Availability

LHS data may be made available to researchers who partner with an LHS researcher, and who apply for data access. The data that support the findings of this study is available on request from the corresponding author.
