# Supplementary material for: Greater temporal regularity of primary care visits was associated with reduced hospitalizations and mortality, even after controlling for continuity of care
Source: BMC Health Serv Res. 2023 Jul 20;23:777. doi: 10.1186/s12913-023-09808-7 (PMC10360299; doi:10.1186/s12913-023-09808-7)
Supplement: Supplementary file 1 — Additional file 1. [file 12913_2023_9808_MOESM1_ESM.docx]

Appendix A: Poisson and Negative Binomial Regression Results

**Table. Poisson regression and negative binomial regression Results to examine the association of TR with number of hospitalization visits.**

|  | With covariates (poisson) | Excluding deaths | Negative binomial |
| --- | --- | --- | --- |
| Quintile 1 | REF | REF | REF |
| Quintile 2 | 1.10 ‡ | 1.09 ‡ | 1.10 ‡ |
| Quintile 3 | 1.16 ‡ | 1.14 ‡ | 1.16 ‡ |
| Quintile 4 | 1.19 ‡ | 1.20 ‡ | 1.20 ‡ |
| Quintile 5 | 1.23 ‡ | 1.21 ‡ | 1.22 ‡ |
| Residual deviance/df | 1.58 | 1.42 |  |

Appendix B: Stratified Analyses

A. Stratified analyses by age

**Table A1. Among patients ages 50-59. Association between temporal regularity of primary care visits and hospitalizations, using logistic regression. TR was measured over a two-year period (2016-2017) and hospitalizations over the following two years (2018-2019).** Total n = 18,010 (of whom, 3,801 with at least one hospitalization)

|  | Range of TR | Percent Hosp. at least once | Odds Ratio –  Unadjusted | Odds Ratio -Adjusted* |
| --- | --- | --- | --- | --- |
| Quintile 1 of TR (most regular) | 0.00-0.71 | 16.4 | REF | REF |
| Quintile 2 | 0.71-0.87 | 21.3 | 1.38 ‡ | 1.20 ‡ |
| Quintile 3 | 0.87-1.02 | 22.5 | 1.49 ‡ | 1.26 ‡ |
| Quintile 4 | 1.02-1.22 | 23.9 | 1.60 ‡ | 1.39 ‡ |
| Quintile 5 (least regular) | 1.22-3.19 | 21.7 | 1.41 ‡ | 1.27 ‡ |

*Adjusted for all variables in Table 2 (age, sex, sector, region, SES, comorbid conditions), and for Bice-Boxerman continuity of care.

† p < 0.05

‡ p < 0.001

**Table A2. Among patients age 60-69. Association between temporal regularity of primary care visits and hospitalizations, using logistic regression. TR was measured over a two-year period (2016-2017) and hospitalizations over the following two years (2018-2019).**

|  | Range of TR | Percent Hosp. at least once | Odds Ratio –  Unadjusted | Odds Ratio -Adjusted* |
| --- | --- | --- | --- | --- |
| Quintile 1 of TR (most regular) | 0.00-0.70 | 21.4 | REF | REF |
| Quintile 2 | 0.70-0.85 | 25.6 | 1.26 ‡ | 1.08 |
| Quintile 3 | 0.85-0.98 | 27.1 | 1.37 ‡ | 1.12 † |
| Quintile 4 | 0.98-1.17 | 26.8 | 1.34 ‡ | 1.11 † |
| Quintile 5 (least regular) | 1.17-2.87 | 27.7 | 1.41 ‡ | 1.20 ‡ |

† p < 0.05

‡ p < 0.001

Total n = 22,773 of whom, 5,858 with at least one hospitalization

**Adjusted for all variables in Table 2 (age, sex, sector, region, SES, comorbid conditions), and for Bice-Boxerman continuity of care.

**Table A3. Among patients age 70-79. Association between temporal regularity of primary care visits and hospitalizations, using logistic regression. TR was measured over a two-year period (2016-2017) and hospitalizations over the following two years (2018-2019).** Total n= 13,667 patients of whom, 5,278 with at least one hospitalization.

|  | Range of TR | Percent Hosp. at least once | Odds Ratio –  Unadjusted | Odds Ratio -Adjusted* |
| --- | --- | --- | --- | --- |
| Quintile 1 of TR (most regular) | 0.00-0.69 | 31.5 | REF | REF |
| Quintile 2 | 0.69-0.82 | 37.8 | 1.32 ‡ | 1.12 |
| Quintile 3 | 0.82-0.95 | 39.5 | 1.42 ‡ | 1.15 † |
| Quintile 4 | 0.95-1.13 | 41.7 | 1.55 ‡ | 1.29 ‡ |
| Quintile 5 (least regular) | 1.13-2.87 | 27.7 | 1.61 ‡ | 1.35 ‡ |

† p < 0.05

‡ p < 0.001

*Adjusted for all variables in Table 2 (age, sex, sector, region, SES, comorbid conditions), and for Bice-Boxerman continuity of care.

**Table A4: Among patients ages 50-59. Association between temporal regularity of primary care visits and mortality, using logistic regression. TR was measured over a two-year period (2016-2017) and mortality over the following two years (2018-2019).** Total n=18,010 of whom, 306 died

|  | Range of TR | Percent mortality | Odds Ratio –  Unadjusted | Odds Ratio -Adjusted* |
| --- | --- | --- | --- | --- |
| Quintile 1 of TR (most regular) | 0.00-0.71 | 1.19 | REF | REF |
| Quintile 2 | 0.71-0.87 | 1.69 | 1.43 | 1.23 |
| Quintile 3 | 0.87-1.02 | 1.75 | 1.47 (p=0.052) | 1.27 |
| Quintile 4 | 1.02-1.22 | 2.00 | 1.69 ‡ | 1.43 |
| Quintile 5 (least regular) | 1.22-3.19 | 1.86 | 1.57 † | 1.35 |

*Adjusted for all variables in Table 2 (age, sex, sector, region, SES, comorbid conditions), and for Bice-Boxerman continuity of care.

† p < 0.05

‡ p < 0.001

**Table A5: Among patients age 60-69. Association between temporal regularity of primary care visits and mortality, using logistic regression. TR was measured over a two-year period (2016-2017) and mortality over the following two years (2018-2019).** Total 22,773 = (of whom, 5,858 died)

|  | Range of TR | Percent mortality | Odds Ratio –  Unadjusted | Odds Ratio -Adjusted* |
| --- | --- | --- | --- | --- |
| Quintile 1 of TR (most regular) | 0.00-0.70 | 2.66 | REF | REF |
| Quintile 2 | 0.70-0.85 | 3.58 | 1.36 † | 1.16 |
| Quintile 3 | 0.85-0.98 | 3.32 | 1.26 | 0.99 |
| Quintile 4 | 0.98-1.17 | 3.78 | 1.44 ‡ | 1.16 |
| Quintile 5 (least regular) | 1.17-2.87 | 4.46 | 1.71 ‡ | 1.40 ‡ |

*Adjusted for all variables in Table 2 (age, sex, sector, region, SES, comorbid conditions), and for Bice-Boxerman continuity of care.

† p < 0.05

‡ p < 0.001

**Table A6. Among patients age 70-79. Association between temporal regularity of primary care visits and mortality, using logistic regression. TR was measured over a two-year period (2016-2017) and mortality over the following two years (2018-2019).**

Total n =13,667 of whom, 1,060 died

|  | Range of TR | Percent mortality | Odds Ratio –  Unadjusted | Odds Ratio -Adjusted* |
| --- | --- | --- | --- | --- |
| Quintile 1 of TR (most regular) | 0.00-0.69 | 5.30 | REF | REF |
| Quintile 2 | 0.69-0.82 | 7.46 | 1.44 ‡ | 1.27 † |
| Quintile 3 | 0.82-0.95 | 8.12 | 1.58 ‡ | 1.31 † |
| Quintile 4 | 0.95-1.13 | 8.16 | 1.59 ‡ | 1.34 † |
| Quintile 5 (least regular) | 1.13-2.87 | 9.73 | 1.92 ‡ | 1.54 ‡ |

† p < 0.05

‡ p < 0.001

*Adjusted for all variables in Table 2 (age, sex, sector, region, SES, comorbid conditions), and for Bice-Boxerman continuity of care.

B. Stratified analysis by sex.

**Table B1: Among female patients. Association between temporal regularity of primary care visits and hospitalizations, using logistic regression. TR was measured over a two-year period (2016-2017) and hospitalizations over the following two years (2018-2019).**

Total n = 35,301 of whom, 9,572 with at least one hospitalization

|  | Range of TR | Percent Hosp. at least once | Odds Ratio –  Unadjusted | Odds Ratio -Adjusted* |
| --- | --- | --- | --- | --- |
| Quintile 1 of TR (most regular) | 0.00-0.69 | 23.9 | REF | REF |
| Quintile 2 | 0.69-0.83 | 28.0 | 1.24 ‡ | 1.10 † |
| Quintile 3 | 0.83-0.97 | 29.4 | 1.33 ‡ | 1.18 ‡ |
| Quintile 4 | 0.97-1.15 | 28.1 | 1.24 ‡ | 1.19 ‡ |
| Quintile 5 (least regular) | 1.15-2.96 | 26.2 | 1.13 † | 1.14 † |

*Adjusted for all variables in Table 2 (age, sex, sector, region, SES, comorbid conditions), and for Bice-Boxerman continuity of care.

† p < 0.05

‡ p < 0.001

**Table B2: Among male patients. Association between temporal regularity of primary care visits and hospitalizations, using logistic regression. TR was measured over a two-year period (2016-2017) and hospitalizations over the following two years (2018-2019).**

Total n= 35,055 of whom, 10,942 with at least one hospitalization

|  | Range of TR | Percent Hosp. at least once | Odds Ratio –  Unadjusted | Odds Ratio -Adjusted* |
| --- | --- | --- | --- | --- |
| Quintile 1 of TR (most regular) | 0.00-0.71 | 26.2 | REF | REF |
| Quintile 2 | 0.71-0.86 | 32.2 | 1.34 ‡ | 1.12 † |
| Quintile 3 | 0.86-1.01 | 32.7 | 1.37 ‡ | 1.14 ‡ |
| Quintile 4 | 1.01-1.21 | 33.1 | 1.39 ‡ | 1.23 ‡ |
| Quintile 5 (least regular) | 1.21-3.19 | 31.8 | 1.32 ‡ | 1.29 ‡ |

*Adjusted for all variables in Table 2 (age, sex, sector, region, SES, comorbid conditions), and for Bice-Boxerman continuity of care.

† p < 0.05

‡ p < 0.001

**Table B3: Among female patients. Association between temporal regularity of primary care visits and mortality, using logistic regression. TR was measured over a two-year period (2016-2017) and mortality over the following two years (2018-2019).**

Total n = 35,301 of whom, 1731 died

|  | Range of TR | Percent mortality | Odds Ratio –  Unadjusted | Odds Ratio -Adjusted* |
| --- | --- | --- | --- | --- |
| Quintile 1 of TR (most regular) | 0.00-0.69 | 4.25 | REF | REF |
| Quintile 2 | 0.69-0.83 | 5.21 | 1.23 † | 1.18 |
| Quintile 3 | 0.83-0.97 | 4.99 | 1.18 † | 1.14 |
| Quintile 4 | 0.97-1.15 | 4.96 | 1.18 † | 1.23 † |
| Quintile 5 (least regular) | 1.15-2.96 | 5.11 | 1.21 † | 1.33 † |

*Adjusted for all variables in Table 2 (age, sex, sector, region, SES, comorbid conditions), and for Bice-Boxerman continuity of care.

† p < 0.05

‡ p < 0.001

**Table B4: Among male patients. Association between temporal regularity of primary care visits and mortality, using logistic regression. TR was measured over a two-year period (2016-2017) and mortality over the following two years (2018-2019).**

Total 35,055 = (of whom, 2,035 died)

|  | Range of TR | Percent mortality | Odds Ratio –  Unadjusted | Odds Ratio -Adjusted* |
| --- | --- | --- | --- | --- |
| Quintile 1 of TR (most regular) | 0.00-0.71 | 4.64 | REF | REF |
| Quintile 2 | 0.71-0.86 | 6.25 | 1.37 ‡ | 1.14 |
| Quintile 3 | 0.86-1.01 | 6.22 | 1.36 ‡ | 1.16 |
| Quintile 4 | 1.01-1.21 | 5.82 | 1.27 † | 1.16 |
| Quintile 5 (least regular) | 1.21-3.19 | 6.10 | 1.34 ‡ | 1.44 ‡ |

*Adjusted for all variables in Table 2 (age, sex, sector, region, SES, comorbid conditions), and for Bice-Boxerman continuity of care.

† p < 0.05

‡ p < 0.001

C. Stratified analyses by sector of society.

**Table C1: Among general population patients. Association between temporal regularity of primary care visits and hospitalizations, using logistic regression. TR was measured over a two-year period (2016-2017) and hospitalizations over the following two years (2018-2019).**

Total n = 54,411 (of whom, 15,722 with at least one hospitalization)

|  | Range of TR | Percent Hosp. at least once | Odds Ratio –  Unadjusted | Odds Ratio -Adjusted* |
| --- | --- | --- | --- | --- |
| Quintile 1 of TR (most regular) | 0.00-0.69 | 24.8 | REF | REF |
| Quintile 2 | 0.69-0.84 | 30.2 | 1.31 ‡ | 1.12 ‡ |
| Quintile 3 | 0.84-0.98 | 30.5 | 1.33 ‡ | 1.15 ‡ |
| Quintile 4 | 0.98-1.17 | 30.0 | 1.30 ‡ | 1.19 ‡ |
| Quintile 5 (least regular) | 1.17-3.19 | 29.0 | 1.24 ‡ | 1.23 ‡ |

*Adjusted for all variables in Table 2 (age, sex, sector, region, SES, comorbid conditions), and for Bice-Boxerman continuity of care.

† p < 0.05

‡ p < 0.001

**Table C2: Among Arab patients. Association between temporal regularity of primary care visits and hospitalizations, using logistic regression. TR was measured over a two-year period (2016-2017) and hospitalizations over the following two years (2018-2019).**

Total n = 11,288 of whom, 3,510 with at least one hospitalization

|  | Range of TR | Percent Hosp. at least once | Odds Ratio –  Unadjusted | Odds Ratio -Adjusted* |
| --- | --- | --- | --- | --- |
| Quintile 1 of TR (most regular) | 0.00-0.73 | 26.5 | REF | REF |
| Quintile 2 | 0.73-0.89 | 31.3 | 1.26 ‡ | 1.10 |
| Quintile 3 | 0.89-1.03 | 32.5 | 1.33 ‡ | 1.12 |
| Quintile 4 | 1.03-1.23 | 33.6 | 1.40 ‡ | 1.23 † |
| Quintile 5 (least regular) | 1.23-3.19 | 31.5 | 1.28 ‡ | 1.18 † |

*Adjusted for all variables in Table 2 (age, sex, sector, region, SES, comorbid conditions), and for Bice-Boxerman continuity of care.

† p < 0.05

‡ p < 0.001

**Table C3: Among general population patients. Association between temporal regularity of primary care visits and mortality, using logistic regression. TR was measured over a two-year period (2016-2017) and mortality over the following two years (2018-2019).**

Total 54,411 = (of whom, 3,070 died)

|  | Range of TR | Percent mortality | Odds Ratio –  Unadjusted | Odds Ratio -Adjusted* |
| --- | --- | --- | --- | --- |
| Quintile 1 of TR (most regular) | 0.00-0.69 | 4.75 | REF | REF |
| Quintile 2 | 0.69-0.84 | 5.97 | 1.27 ‡ | 1.14 † |
| Quintile 3 | 0.84-0.98 | 5.96 | 1.27 ‡ | 1.14 † |
| Quintile 4 | 0.98-1.17 | 5.66 | 1.20 † | 1.16 † |
| Quintile 5 (least regular) | 1.17-3.19 | 5.86 | 1.25 ‡ | 1.33 ‡ |

*Adjusted for all variables in Table 2 (age, sex, sector, region, SES, comorbid conditions), and for Bice-Boxerman continuity of care.

† p < 0.05

‡ p < 0.001

**Table C4: Among Arab patients. Association between temporal regularity of primary care visits and mortality, using logistic regression. TR was measured over a two-year period (2016-2017) and mortality over the following two years (2018-2019).**

Total n = 11,288 of whom, 483 died

|  | Range of TR | Percent mortality | Odds Ratio –  Unadjusted | Odds Ratio -Adjusted* |
| --- | --- | --- | --- | --- |
| Quintile 1 of TR (most regular) | 0.00-0.73 | 3.01 | REF | REF |
| Quintile 2 | 0.73-0.89 | 4.21 | 1.41 † | 1.22 |
| Quintile 3 | 0.89-1.03 | 4.07 | 1.37 (0.05) | 1.11 |
| Quintile 4 | 1.03-1.23 | 4.70 | 1.59 † | 1.29 |
| Quintile 5 (least regular) | 1.23-3.19 | 5.41 | 1.84 ‡ | 1.75 ‡ |

*Adjusted for all variables in Table 2 (age, sex, sector, region, SES, comorbid conditions), and for Bice-Boxerman continuity of care.

† p < 0.05

‡ p < 0.001

D. Stratified analyses by area-level socioeconomic status

**Table D1: Among poorest patients (SES 1-3). Association between temporal regularity of primary care visits and hospitalizations, using logistic regression. TR was measured over a two-year period (2016-2017) and hospitalizations over the following two years (2018-2019).**

Total n = 11,270 of whom, 3,343 with at least one hospitalization

|  | Range of TR | Percent Hosp. at least once | Odds Ratio –  Unadjusted | Odds Ratio -Adjusted* |
| --- | --- | --- | --- | --- |
| Quintile 1 of TR (most regular) | 0.00-0.72 | 25.0 | REF | REF |
| Quintile 2 | 0.72-0.87 | 29.3 | 1.24 † | 1.08 |
| Quintile 3 | 0.87-1.01 | 30.8 | 1.33 ‡ | 1.16 † |
| Quintile 4 | 1.01-1.21 | 33.4 | 1.50 ‡ | 1.43 ‡ |
| Quintile 5 (least regular) | 1.21-2.82 | 29.9 | 1.28 ‡ | 1.25 † |

*Adjusted for all variables in Table 2 (age, sex, sector, region, SES, comorbid conditions), and for Bice-Boxerman continuity of care.

† p < 0.05

‡ p < 0.001

**Table D2: Among poor patients (SES 4-5) Association between temporal regularity of primary care visits and hospitalizations, using logistic regression. TR was measured over a two-year period (2016-2017) and hospitalizations over the following two years (2018-2019).**

Total n = 30,419 of whom, 9,098 with at least one hospitalization

|  | Range of TR | Percent Hosp. at least once | Odds Ratio –  Unadjusted | Odds Ratio -Adjusted* |
| --- | --- | --- | --- | --- |
| Quintile 1 of TR (most regular) | 0.00-0.71 | 25.5 | REF | REF |
| Quintile 2 | 0.71-0.85 | 31.0 | 1.31 ‡ | 1.16 ‡ |
| Quintile 3 | 0.85-0.99 | 32.6 | 1.41 ‡ | 1.22 ‡ |
| Quintile 4 | 0.99-1.18 | 31.0 | 1.31 ‡ | 1.19 ‡ |
| Quintile 5 (least regular) | 1.18-3.19 | 29.4 | 1.21 ‡ | 1.19 ‡ |

*Adjusted for all variables in Table 2 (age, sex, sector, region, SES, comorbid conditions), and for Bice-Boxerman continuity of care.

† p < 0.05

‡ p < 0.001

**Table D3. Among subgroup 3: SES 6-7. Association between temporal regularity of primary care visits and hospitalizations, using logistic regression. TR was measured over a two-year period (2016-2017) and hospitalizations over the following two years (2018-2019).**

Total n = 22,124 of whom, 6,265 with at least one hospitalization

|  | Range of TR | Percent Hosp. at least once | Odds Ratio –  Unadjusted | Odds Ratio -Adjusted* |
| --- | --- | --- | --- | --- |
| Quintile 1 of TR (most regular) | 0.00-0.69 | 24.7 | REF | REF |
| Quintile 2 | 0.69-0.83 | 29.8 | 1.30 ‡ | 1.06 |
| Quintile 3 | 0.83-0.97 | 28.6 | 1.22 ‡ | 1.01 |
| Quintile 4 | 0.97-1.16 | 29.3 | 1.27 ‡ | 1.10 |
| Quintile 5 (least regular) | 1.16-2.96 | 29.1 | 1.25 ‡ | 1.21 ‡ |

*Adjusted for all variables in Table 2 (age, sex, sector, region, SES, comorbid conditions), and for Bice-Boxerman continuity of care.

† p < 0.05

‡ p < 0.001

**Table D4. Among subgroup 4: SES 8-10. Association between temporal regularity of primary care visits and hospitalizations, using logistic regression. TR was measured over a two-year period (2016-2017) and hospitalizations over the following two years (2018-2019).**

Total n = 5921 (of whom, 1631 with at least one hospitalization)

|  | Range of TR | Percent Hosp. at least once | Odds Ratio –  Unadjusted | Odds Ratio -Adjusted* |
| --- | --- | --- | --- | --- |
| Quintile 1 of TR (most regular) | 0.00-0.68 | 23.3 | REF | REF |
| Quintile 2 | 0.68-0.82 | 29.2 | 1.36 ‡ | 1.16 |
| Quintile 3 | 0.82-0.96 | 28.1 | 1.29 ‡ | 1.10 |
| Quintile 4 | 0.96-1.15 | 29.3 | 1.37 ‡ | 1.24 † |
| Quintile 5 (least regular) | 1.15-2.75 | 27.8 | 1.27 † | 1.18 |

*Adjusted for all variables in Table 2 (age, sex, region, SES, comorbid conditions), and for Bice-Boxerman continuity of care. Analyses were not adjusted for sector because almost all the patients were from the general population.

† p < 0.05

‡ p < 0.001

.

**Table D5: Among poorest patients (SES 1-3). Association between temporal regularity of primary care visits and mortality, using logistic regression. TR was measured over a two-year period (2016-2017) and mortality over the following two years (2018-2019).**

Total n = 11,270 of whom, 530 died

|  | Range of TR | Percent mortality | Odds Ratio –  Unadjusted | Odds Ratio -Adjusted* |
| --- | --- | --- | --- | --- |
| Quintile 1 of TR (most regular) | 0.00-0.72 | 3.64 | REF | REF |
| Quintile 2 | 0.72-0.87 | 4.48 | 1.24 | 1.06 |
| Quintile 3 | 0.87-1.01 | 4.70 | 1.31 | 1.14 |
| Quintile 4 | 1.01-1.21 | 5.32 | 1.49 † | 1.45 † |
| Quintile 5 (least regular) | 1.21-2.82 | 5.37 | 1.50 † | 1.63 † |

*Adjusted for all variables in Table 2 (age, sex, sector, region, SES, comorbid conditions), and for Bice-Boxerman continuity of care.

† p < 0.05

‡ p < 0.001

**Table D6: Among poor patients (SES 4-5). Association between temporal regularity of primary care visits and mortality, using logistic regression. TR was measured over a two-year period (2016-2017) and mortality over the following two years (2018-2019).**

Total n = 30,419 of whom, 1,682 died

|  | Range of TR | Percent mortality | Odds Ratio –  Unadjusted | Odds Ratio -Adjusted* |
| --- | --- | --- | --- | --- |
| Quintile 1 of TR (most regular) | 0.00-0.71 | 4.65 | REF | REF |
| Quintile 2 | 0.71-0.85 | 5.75 | 1.25 † | 1.14 |
| Quintile 3 | 0.85-0.99 | 6.13 | 1.34 ‡ | 1.20 † |
| Quintile 4 | 0.99-1.18 | 5.59 | 1.21 † | 1.18 |
| Quintile 5 (least regular) | 1.18-3.19 | 5.52 | 1.20 † | 1.31† |

*Adjusted for all variables in Table 2 (age, sex, sector, region, SES, comorbid conditions), and for Bice-Boxerman continuity of care.

† p < 0.05

‡ p < 0.001

**Table D7. Among subgroup 3: SES 6-7. Association between temporal regularity of primary care visits and mortality, using logistic regression. TR was measured over a two-year period (2016-2017) and mortality over the following two years (2018-2019).**

Total n = 22,124 (of whom, 1,160 died)

|  | Range of TR | Percent mortality | Odds Ratio –  Unadjusted | Odds Ratio -Adjusted* |
| --- | --- | --- | --- | --- |
| Quintile 1 of TR (most regular) | 0.00-0.69 | 4.56 | REF | REF |
| Quintile 2 | 0.69-0.83 | 5.76 | 1.28 † | 1.03 |
| Quintile 3 | 0.83-0.97 | 4.97 | 1.09 | 0.89 |
| Quintile 4 | 0.97-1.16 | 5.24 | 1.16 | 0.98 |
| Quintile 5 (least regular) | 1.16-2.96 | 5.67 | 1.26 † | 1.21 |

*Adjusted for all variables in Table 2 (age, sex, sector, region, SES, comorbid conditions), and for Bice-Boxerman continuity of care.

† p < 0.05

‡ p < 0.001

**Table D8. Among subgroup 4: SES 8-10. Association between temporal regularity of primary care visits and mortality, using logistic regression. TR was measured over a two-year period (2016-2017) and mortality over the following two years (2018-2019).**

Total n =5,921 of whom, 371 died

|  | Range of TR | Percent mortality | Odds Ratio –  Unadjusted | Odds Ratio -Adjusted* |
| --- | --- | --- | --- | --- |
| Quintile 1 of TR (most regular) | 0.00-0.68 | 3.97 | REF | REF |
| Quintile 2 | 0.68-0.82 | 7.43 | 1.94 ‡ | 1.69 ‡ |
| Quintile 3 | 0.82-0.96 | 6.67 | 1.73 ‡ | 1.47(p=0.053) |
| Quintile 4 | 0.96-1.15 | 6.76 | 1.75 ‡ | 1.59 † |
| Quintile 5 (least regular) | 1.15-2.75 | 27.8 | 1.68 ‡ | 1.49 † |

*Adjusted for all variables in Table 2 (age, sex, region, SES, comorbid conditions), and for Bice-Boxerman continuity of care.

† p < 0.05

‡ p < 0.001

E. Stratified analyses by particular comorbid conditions

**Table E1: Among patients with depression. Association between temporal regularity of primary care visits and hospitalizations, using logistic regression. TR was measured over a two-year period (2016-2017) and hospitalizations over the following two years (2018-2019).**

Total n = 20,200 (of whom, 7,594 with at least one hospitalization)

|  | Range of TR | Percent Hosp. at least once | Odds Ratio –  Unadjusted | Odds Ratio -Adjusted* |
| --- | --- | --- | --- | --- |
| Quintile 1 of TR (most regular) | 0.00-0.71 | 33.5 | REF | REF |
| Quintile 2 | 0.71-0.84 | 38.3 | 1.23 ‡ | 1.09 |
| Quintile 3 | 0.84-0.97 | 37.4 | 1.19 ‡ | 1.07 |
| Quintile 4 | 0.97-1.15 | 38.9 | 1.26 ‡ | 1.19 ‡ |
| Quintile 5 (least regular) | 1.15-3.19 | 40.0 | 1.32 ‡ | 1.29 ‡ |

*Adjusted for all variables in Table 2 (age, sex, sector, region, SES, comorbid conditions), and for Bice-Boxerman continuity of care.

† p < 0.05

‡ p < 0.001

**Table E2: Among patients with moderate or severe chronic kidney disease. Association between temporal regularity of primary care visits and hospitalizations, using logistic regression. TR was measured over a two-year period (2016-2017) and hospitalizations over the following two years (2018-2019).**

Total n=5,763 of whom, 3,443 with at least one hospitalization

|  | Range of TR | Percent Hosp. at least once | Odds Ratio –  Unadjusted | Odds Ratio -Adjusted* |
| --- | --- | --- | --- | --- |
| Quintile 1 of TR (most regular) | 0.00-0.72 | 53.3 | REF | REF |
| Quintile 2 | 0.72-0.85 | 59.4 | 1.28 † | 1.13 |
| Quintile 3 | 0.85-0.98 | 61.6 | 1.41 ‡ | 1.25 † |
| Quintile 4 | 0.98-1.16 | 61.2 | 1.38 ‡ | 1.22 † |
| Quintile 5 (least regular) | 1.16-2.87 | 63.3 | 1.51 ‡ | 1.38 ‡ |

*Adjusted for all variables in Table 2 (age, sex, sector, region, SES, comorbid conditions), and for Bice-Boxerman continuity of care.

† p < 0.05

‡ p < 0.001

**Table E3: Among patients with cancer. Association between temporal regularity of primary care visits and hospitalizations, using logistic regression. TR was measured over a two-year period (2016-2017) and hospitalizations over the following two years (2018-2019).**

Total n = 7,908 of whom, 3,070 with at least one hospitalization

|  | Range of TR | Percent Hosp. at least once | Odds Ratio –  Unadjusted | Odds Ratio -Adjusted* |
| --- | --- | --- | --- | --- |
| Quintile 1 of TR (most regular) | 0.00-0.70 | 34.8 | REF | REF |
| Quintile 2 | 0.70-0.83 | 40.4 | 1.27 † | 1.13 |
| Quintile 3 | 0.83-0.95 | 40.3 | 1.26 † | 1.09 |
| Quintile 4 | 0.95-1.12 | 40.3 | 1.27 † | 1.15 |
| Quintile 5 (least regular) | 1.12-2.96 | 38.4 | 1.17 † | 1.05 |

*Adjusted for all variables in Table 2 (age, sex, sector, region, SES, comorbid conditions), and for Bice-Boxerman continuity of care.

† p < 0.05

‡ p < 0.001

**Table E4: Among patients with depression. Association between temporal regularity of primary care visits and mortality, using logistic regression. TR was measured over a two-year period (2016-2017) and mortality over the following two years (2018-2019).**

Total n = 20,200 of whom, 1,530 died

|  | Range of TR | Percent mortality | Odds Ratio –  Unadjusted | Odds Ratio -Adjusted* |
| --- | --- | --- | --- | --- |
| Quintile 1 of TR (most regular) | 0.00-0.71 | 6.49 | REF | REF |
| Quintile 2 | 0.71-0.84 | 8.29 | 1.30 † | 1.26 † |
| Quintile 3 | 0.84-0.97 | 7.03 | 1.09 | 1.04 |
| Quintile 4 | 0.97-1.15 | 7.62 | 1.19 † | 1.19 |
| Quintile 5 (least regular) | 1.15-3.19 | 8.44 | 1.33 ‡ | 1.41 ‡ |

*Adjusted for all variables in Table 2 (age, sex, sector, region, SES, comorbid conditions), and for Bice-Boxerman continuity of care.

† p < 0.05

‡ p < 0.001

**Table E5: Among patients with moderate or severe chronic kidney disease. Association between temporal regularity of primary care visits and mortality, using logistic regression. TR was measured over a two-year period (2016-2017) and mortality over the following two years (2018-2019).**

Total n= 5,763 of whom, 1,204 died

|  | Range of TR | Percent mortality | Odds Ratio –  Unadjusted | Odds Ratio -Adjusted* |
| --- | --- | --- | --- | --- |
| Quintile 1 of TR (most regular) | 0.00-0.72 | 20.2 | REF | REF |
| Quintile 2 | 0.72-0.85 | 21.6 | 1.09 | 1.05 |
| Quintile 3 | 0.85-0.98 | 21.7 | 1.09 | 1.08 |
| Quintile 4 | 0.98-1.16 | 19.1 | 0.93 | 0.90 |
| Quintile 5 (least regular) | 1.16-2.87 | 21.9 | 1.11 | 1.12 |

*Adjusted for all variables in Table 2 (age, sex, sector, region, SES, comorbid conditions), and for Bice-Boxerman continuity of care.

† p < 0.05

‡ p < 0.001

**Table E6: Among patients with cancer. Association between temporal regularity of primary care visits and mortality, using logistic regression. TR was measured over a two-year period (2016-2017) and mortality over the following two years (2018-2019).**

Total n = 7,908 of whom, 853 died

|  | Range of TR | Percent mortality | Odds Ratio –  Unadjusted | Odds Ratio -Adjusted* |
| --- | --- | --- | --- | --- |
| Quintile 1 of TR (most regular) | 0.00-0.70 | 8.79 | REF | REF |
| Quintile 2 | 0.70-0.83 | 11.4 | 1.34 † | 1.33 † |
| Quintile 3 | 0.83-0.95 | 10.9 | 1.27 † | 1.22 |
| Quintile 4 | 0.95-1.12 | 11.0 | 1.28 † | 1.26 |
| Quintile 5 (least regular) | 1.12-2.96 | 11.8 | 1.38 † | 1.32 † |

*Adjusted for all variables in Table 2 (age, sex, sector, region, SES, comorbid conditions), and for Bice-Boxerman continuity of care.

† p < 0.05

‡ p < 0.001
